# *Strongyloides* Hyperinfection Syndrome among COVID-19 Patients Treated with Corticosteroids

**DOI:** 10.3201/eid2807.220198

**Published:** 2022-07

**Authors:** Jani M. Kim, Geetha Sivasubramanian

**Affiliations:** University of California San Francisco, Fresno, California, USA

**Keywords:** parasites, Strongyloides stercoralis, COVID-19, respiratory infections, Strongyloides, severe acute respiratory syndrome coronavirus 2, SARS-CoV-2, SARS, coronavirus disease, zoonoses, viruses, coronavirus

## Abstract

Widespread use of corticosteroids for COVID-19 treatment has led to *Strongyloides* reactivation and severe disease in patients from endemic areas. We describe a US patient with COVID-19 and *Strongyloides* hyperinfection syndrome and review other reported cases. Our findings highlight the need for *Strongyloides* screening and treatment in high-risk populations.

Strongyloidiasis is caused by the soil-transmitted helminth *Strongyloides stercoralis* and affects ≈613.8 million persons worldwide (*1*). *S. stercoralis* infections can be asymptomatic or chronic or can cause life-threatening larva dissemination, especially in immunocompromised patients (*2*). 

Among COVID-19 patients, dexamethasone is the standard treatment for persons requiring supplemental oxygen, but among persons from *Strongyloides*-endemic areas, exposure to corticosteroids can cause life-threatening *S. stercoralis* hyperinfection (*3*). We describe a case of *Strongyloides* hyperinfection syndrome in a COVID-19 patient and review other reported cases. 

A 63-year-old man, who was originally from Cambodia, was admitted to a hospital in Central Valley, California, USA, for a 4-day history of fever, cough, and respiratory distress. His medical history included diabetes mellitus and alcohol use disorder. Admission laboratory testing showed a leukocyte count of 8,500 cells/μL (absolute lymphocyte count 660 cells/μL, reference range 800–4,800 cells/μL) and absolute eosinophil count of 0 cells/μL (reference range 0–800 cells/μL). A nasopharyngeal swab sample tested SARS-CoV-2–positive by PCR. Chest radiographs showed patchy bilateral airspace consolidations. By day 5 of hospitalization, the patient’s respiratory failure worsened, and he required supplemental oxygen via high-flow nasal cannula. Chest computed tomography imaging showed multifocal bilateral airspace opacities. The patient received intravenous dexamethasone (6 mg/d for 10 d); during the first 5 days of treatment, he also received baricitinib (10 mg 1×/d) and remdesivir (100 mg/d). The patient’s respiratory status improved, and he was discharged to a skilled nursing facility.

The patient returned to the hospital 6 days later with respiratory failure and altered mental status. He was febrile (temperature 39°C) and hypoxic and required intubation. Blood tests revealed a leukocyte of 5,300 cells/μL (absolute lymphocyte count 1,000 cells/μL) and absolute eosinophil count of 100 cells/μL. Blood and sputum cultures were positive for methicillin-sensitive *Staphylococcus aureus*, and we initiated intravenous cefazolin (2 g every 8 h for 10 d). The patient transiently improved, but then fever developed and persisted. After 10 days of broad-spectrum antimicrobial drug therapy, the patient’s blood cultures were negative. Echocardiography, magnetic resonance imaging, and computed tomography scans did not identify a focus of infection.

Because of the patient’s continued fever and worsening respiratory failure, we performed a diagnostic bronchoscopy on day 28 of his illness. Microscopic examination of the bronchoalveolar lavage fluid revealed parasitic worms consistent with *Strongyloides* spp*.* (Figure). Stool samples were negative for parasites, but *Strongyloides* serum IgG was positive. The patient’s absolute eosinophil count increased to 1,500 cells/μL, and we began oral ivermectin (200 µg/kg for 14 d).

**Figure:**
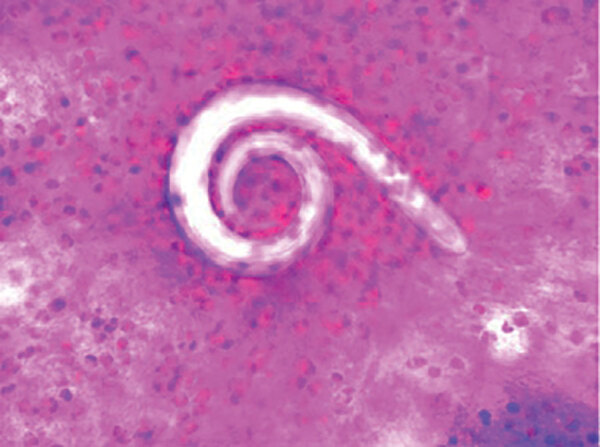
Bronchoalveolar lavage sample showing larval forms of *Strongyloides* *stercoralis* in a patient with COVID-19, United States. Original magnification ×200.

Subsequent respiratory culture was positive for extended spectrum β-lactamase *Escherichia coli*. The patient continued to have encephalopathy, and we recommended a lumbar puncture, but the procedure was not performed because of his hemodynamic instability. We changed the patient’s therapy to intravenous meropenem (2 g every 8 h), but his condition did not improve. He was eventually transitioned to comfort care and died.

*S. stercoralis* parasites are endemic in tropical and subtropical regions, but data on strongyloidiasis prevalence is likely underreported, even in endemic areas (*1*). Patients can develop chronic *S. stercoralis* infection, but an immunocompetent host’s immune system can regulate infection by controlling adult worm population density in the intestines. However, when a host becomes immunocompromised, larval migration to organs can increase during the autoinfection cycle, causing *Strongyloides* hyperinfection syndrome. Exposure to corticosteroids, human T-cell leukemia virus type 1 co-infection, and solid organ transplantation can increase risk for *Strongyloides* hyperinfection syndrome (*2*). Dexamethasone is the standard treatment for COVID-19 patients who require oxygen therapy; other immunosuppressive agents, including interleukin-6 inhibitors such as tocilizumab, also are commonly used. 

Other strongyloidiasis cases have been reported in COVID-19 patients (*4*–*9*) (Table). *Strongyloides* hyperinfection syndrome can cause signs and symptoms similar to those of severe COVID-19, including fever, chills, dyspnea, gastrointestinal symptoms, and rash. These vague symptoms can cause missed or delayed strongyloidiasis diagnosis, demonstrating the need for increased awareness of this condition and systematic screening of high-risk patients.

**Table:** Characteristics of previously reported *Strongyloides* infections in patients with SARS-CoV-2 pneumonia*

Ref no.	Patient age, y/sex	Reporting country	Country of origin	COVID-19 treatment	*Strongyloides*		Eosinophil pattern
					Diagnosis	Treatment	
(*3*)	59/M	Belgium	Ecuador	Anakinra, methylprednisolone 80 mg tapered over 1 month	Positive serologic test; RT-PCR positive for *S. stercoralis* in fecal samples	Single dose ivermectin	Initial eosinopenia (0 cells/mL), elevated to 2,670 cells/mL after steroid exposure, decreased after ivermectin
(*4*)	68/M	United States	Ecuador	Tocilizumab ×1 d and methylprednisolone ×8 d	Sputum culture positive for larvae; positive *Strongyloides* IgG serology	Ivermectin and albendazole ×2 wk	Initial eosinopenia (0 cells/mL), elevated to 1,900 cells/mL after steroid exposure, decreased to 900 cells/mL after ivermectin
(*5*)	59/M	Italy	Southern Italy	Hydroxychloroquine, lopinavir/ritonavir, tocilizumab ×2 d, dexamethasone ×11 d	Stool microscopy positive for rhabditiform larvae; serology positive at 1:640	Oral ivermectin ×4 d	Elevated to 5,540 cells/μL after steroid exposure, rapid decrease after ivermectin
(*6*)	53/M	India	Central India	Methylprednisolone 60 mg intravenous 2×/d ×5 d	Stool microscopy positive for rhabditiform larvae of *S. stercoralis*	Ivermectin and albendazole ×2 wk	Unremarkable
(*7*)	69/M	Spain	Colombia	Methylprednisolone	Bronchoalveolar fluid positive for larvae	Oral ivermectin ×2 wk	Unremarkable
(*8*)	44/M	Spain	Bolivia	Dexamethasone	Positive ELISA IgG serology, 2.27†	Oral ivermectin ×2 d	Eosinopenia before treatment, no further report
	74/F	Spain	Honduras	Dexamethasone	Positive ELISA IgG serology, 2.47†	Oral ivermectin ×2 d	Eosinopenia before treatment, no further report

*All patients recovered. Ref, reference; RT-PCR, reverse transcription PCR. †Normal value <1.01.

Algorithms to aid clinicians with risk assessment, screening, and treatment for *Strongyloides* infection in COVID-19 patients have been proposed (*10*). *Strongyloides* hyperinfection syndrome should be included in the differential diagnosis for patients from endemic areas who receive dexamethasone for COVID-19 and experience clinical decompensation, especially with gram-negative rod bacteremia, pneumonia, or meningitis. Serologic testing should be performed simultaneously and should not delay treatment. Presumptive oral ivermectin for 1–2 days can be considered for COVID-19 patients with higher risk for strongyloidiasis who need dexamethasone (*10*).

Chronic peripheral eosinophilia can be a marker for prompt *Strongyloides* screening. Several case studies have shown a pattern of initial eosinopenia in patients with chronic strongyloidiasis and COVID-19 suppressed with corticosteroids (*4*–*6*). Eosinophils became elevated in these patients because *Strongyloides* hyperinfection developed after corticosteroid administration. In some cases, eosinophilia improved with ivermectin treatment.

In conclusion, *Strongyloides* hyperinfection cases are rising in certain COVID-19 patients. Standardized protocols for *Strongyloides* screening and treatment are needed, especially for patients from endemic countries. To prevent this complication, clinicians should consider *Strongyloides* screening in COVID-19 patients from endemic areas who require corticosteroid treatment.
